# Spontaneous Pneumomediastinum in Labor

**DOI:** 10.1155/2017/6235076

**Published:** 2017-02-21

**Authors:** Mohamed Adnane Berdai, Said Benlamkadem, Smael Labib, Mustapha Harandou

**Affiliations:** Obstetric and Pediatric Intensive Care Unit, Hassan II University Hospital, Sidi Mohamed Ben Abdellah University, Fez, Morocco

## Abstract

Spontaneous pneumomediastinum and subcutaneous emphysema also known as Hamman's syndrome is a very rare complication of labor that is often related to the valsalva maneuver during the labor. In most case, Hamman's syndrome is a self-limiting condition, rarely complicated unless there are underlying respiratory diseases. Chest X-ray can be a useful early diagnostic technique in severe clinical presentation. We report an uneventful pregnancy in a primigravid parturient, which was complicated in the late second stage of labor by the development of subcutaneous emphysema, pneumomediastinum, and mild pneumothorax. Spontaneous recovery occurred after four days of conservative management. This condition shows the major interest of labor analgesia especially locoregional techniques.

## 1. Introduction

Labor related pneumomediastinum and subcutaneous emphysema is also known as Hamman's syndrome, it is named after the physician who first described pneumomediastinum in association with subcutaneous emphysema during pregnancy in 1945 [1]. We report the case of an uneventful pregnancy in a primigravid parturient, which was complicated in the late second stage of labor by the development of Hamman's syndrome.

## 2. Case Report

A 22-year-old nulliparous, with no medical history, presented to our hospital in established labor after a spontaneous rupture of membranes, at 40 weeks of pregnancy. The first stage of labor lasted for 6 hours. During labor, she refused to receive any form of analgesia. At the end of the second stage of labor which had lasted for 2 hours, the patient began to cough; she also complained of shortness of breath and pain over the chest and the neck regions.

Physical examination revealed conscious patient; the blood pressure was 120/90 mmHg, pulse was 96/min, and the oxygen saturation was 98% on 3 L/min of oxygen. The patient had mild dyspnea with breath rate of 24 cycles/minutes. No edema was present and the patient was afebrile; the cardiopulmonary auscultation was normal.

**Figure 1:**
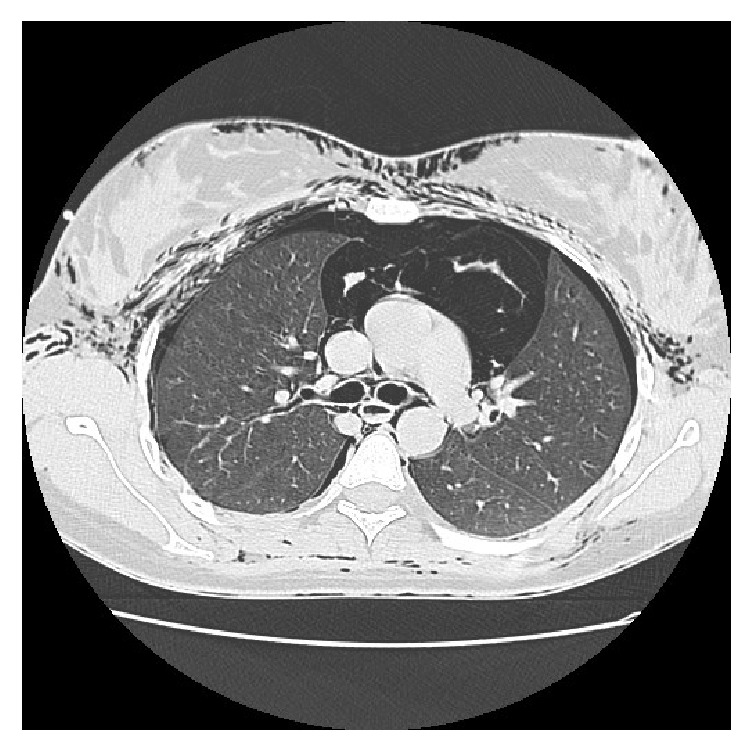
Thoracic computed tomography showing pneumomediastinum, subcutaneous emphysema, and minimal pneumothorax.

**Figure 2:**
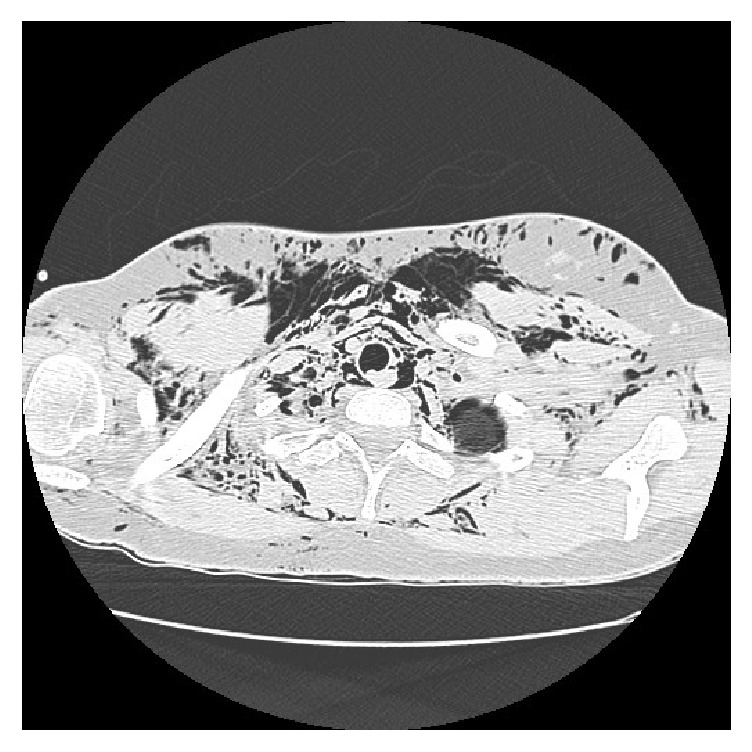
Cervical computed tomography showing extension of subcutaneous emphysema to the cervical region.

Palpation showed crepitus underneath the skin of the anterior neck and bilateral subclavian regions consistent with subcutaneous emphysema. The patient had not undergone any radiological examination due to the imminent delivery and the stability of the respiratory function. She received oxygen by facial mask and remained stable. 15 minutes later, the labor ended with a vaginal delivery requiring an episiotomy. The patient delivered a healthy, live infant with Apgar score of 10/10 in the first and fifth minute. One hour later, the cripitations extended to the upper anterior chest wall and to the face including the eyelids without any deterioration in respiratory status. A chest X-ray showed pneumomediastinum with subcutaneous emphysema extending to the soft tissue of the axillary region. Computed tomography (Figures 1 and 2) confirmed pneumomediastinum and subcutaneous emphysema extending to the thoracic, cervical, and abdominal regions. It showed also a minimal pneumothorax. There were no anomalies in the pulmonary parenchyma and thoracic great vessels. Routine blood tests and arterial gas were found to be normal.

The patient had been followed up for 4 days in hospital, without a specific medication. The symptoms have regressed and chest X-ray showed disappearance of pneumomediastinum. The patient had been discharged home. Regular follow-up showed complete recovery without any sequelae.

## 3. Discussion

The estimated incidences of subcutaneous emphysema and pneumomediastinum in labor and delivery are between 1/2000 and 1/10000 [2]. The pathophysiology involves rupture of marginal alveoli into perivascular tissue planes, with trapping of air into the mediastinum [3]. It is related to the valsalva maneuver during the labor that increases intrathoracic pressure associated with coughing, vomiting, screaming, or pushing in labor. The increased intrathoracic pressure, combined with decreased vascular calibre, establishes a pressure gradient into the vascular sheath, where air can dissect into the mediastinum and from there into the subcutaneous tissues along fascial planes [4]. Spontaneous pneumomediastinum can be seen in situations other than Hamman's syndrome: forceful coughing related to asthmatic bronchospasm or infections, physical activity, and vomiting [5].

Despite being unproved, some suggest that Hamman's syndrome is most likely to occur in a long labor [6]. However, there was no evidence to substantiate this. A large analysis of 187 cases of Hamman's syndrome, with and without evidence of pneumothorax, revealed that 95% of women were primiparous and both first and second stages of labor were of normal duration. Average fetal size was also found to be within normal limits [7].

The onset of symptoms of pregnancy-related pneumomediastinum usually develops during labor; however, clinical appearance is often delayed until the postpartum phase [8]. These symptoms have also been reported in the earlier antepartum period, during hyperemesis or even spontaneously at rest [9]. The symptoms include chest pain, dysphagia, dysphonia, dyspnea, cough, palpitations, anxiety, and hemoptysis [10]. A chest X-ray usually reveals the subcutaneous emphysema, and significant pneumomediastinum or pneumopericardium may be visible.

In our case, the patient was primiparous and had regular length of the first and second stages of labor. She delivered with an important pushing effort as she had not any form of analgesia.

Pneumothorax occurs due to a sudden rise in mediastinal pressure with rupture of the parietal mediastinal pleura. It is reported in a third of cases [11]. However, an isolated pneumothorax can occur in patients with predisposing lung disease.

The importance of awareness of this condition, which is usually self-limiting, is to be able to distinguish it from other more life-threatening postpartum conditions such as pulmonary embolism, amniotic fluid embolism, aortic dissection, and myocardial infarction [10].

Hamman's syndrome is a self-limiting condition, rarely complicated, unless there are underlying respiratory diseases. In the absence of pneumothorax that can cause cardiorespiratory collapse, Hamman's syndrome can resolve with bed rest or even with a conservative treatment within 2 weeks [12]. Treatment is supportive and based on administration of oxygen and provision of analgesia. Management includes avoidance of factors likely to exacerbate the condition such as active pushing, the use of nitric oxide and positive pressure ventilation, and attention to the reassurance and comfort of the patient [13]. Regional anaesthesia is preferred for caesarean section, in order to avoid positive pressure ventilation [14].

## 4. Conclusion 

Hamman's syndrome is a rare complication of labor. It is often a benign condition that is treated conservatively and most cases have a self-limiting course. This condition shows the major interest of labor analgesia especially locoregional techniques.
